# Tasty but Nasty? The Moderating Effect of Message Appeals on Food Neophilia/Neophobia as a Personality Trait: A Case Study of Pig Blood Cake and Meatballs

**DOI:** 10.3390/foods10051093

**Published:** 2021-05-14

**Authors:** Yen-Cheng Chen, Ching-Sung Lee, Shuo-Hui Kuan

**Affiliations:** 1 Department of Applied Science of Living, Chinese Culture University, Taipei 11114, Taiwan; CYC4@g.pccu.edu.tw; 2 Department of Restaurant, Hotel and Institutional Management, Fu Jen Catholic University, New Taipei City 242062, Taiwan; 499246019@mail.fju.edu.tw

**Keywords:** food tourism, rice-based delicacies, food neophilia, food neophobia, personality trait

## Abstract

Given the development of food tourism, food culture has become an important motivation for tourists. This study focuses on food tourism and examines the effects of message appeal and personality traits (food neophilia or neophobia) on tourists’ willingness to consume pig blood cake (PBC) and meatballs, two rice-based Taiwanese street foods. A total of 181 valid questionnaires were administered to foreign tourists in Taiwan (the majority of subjects were Europeans and Americans) through snowball sampling. The questionnaires were analysed using the AMOS 6.0 statistical software package. Foreign tourists’ food neophobia or neophilia was found to significantly affect their willingness to consume rice-based Taiwanese street food (PBC and meatballs) and to strongly regulate the effect of message appeal on their willingness to consume the two delicacies. Past studies on food neophobia/neophilia traits have mostly focused on Western and European foods and have rarely investigated the effect of message appeal on the consumption of traditional rice-based street food in Eastern Asia (e.g., Taiwanese special delicacies). This study’s most important contribution is that food neophilia or neophobia moderates the message appeal effect on foreign tourists’ intention to consume local delicacies. This finding has implications for the hospitality industry and relevant government agencies in Asia for the marketing and promotion of food tourism.

## 1. Introduction

Past studies have shown that language and cultural differences are the greatest barriers between catering operators and tourists. These differences form the basis on which tourists assess the quality of the food they consume, which in turn affects their willingness to eat some foods [1]. Studies have also found that information appeal can persuade consumers to purchase goods or services, affect their attitudes and emotions through aroused interest in products or services and ultimately trigger specific purchase behaviours [2,3]. For some tourists, the lure of experiencing a food culture vastly different from their own guides them to understand the local culture through tasting and preparing food and through the dining environment and atmosphere. These activities can provide a new and profound experience and lasting good memories of the tourism destination, which intensify their feelings about the destination [4,5,6,7]. In keeping with the world’s food tourism trends and to promote Taiwan’s international image, in recent years, Taiwanese government agencies have frequently used Taiwanese cuisine as a diplomatic tool and tourism promotion strategy [8]. Through this strategy, Taiwanese delicacies serve as the focus of marketing because of their strong Taiwanese characteristics, and international exchange activities, international sales meetings, international media interviews and marketing promotion are used to increase the international visibility of Taiwanese cuisine.

As indicated by media reports in recent years, Taiwanese cuisine is quite attractive [9,10]. However, international media have acted as a double-edged sword, making some Taiwanese street food (e.g., pig blood cake (PBC) and preserved eggs) “notorious”, which in turn affects foreigners’ overall perception of Taiwanese delicacies. Language differences are the greatest barrier between catering operators and travellers; these differences form the basis on which travellers evaluate the quality of the food they consume, which in turn affects their willingness to consume it [11,12,13]. Because the root cause of unwillingness to consume new foods is language and cultural differences, it has been postulated that foreigners’ uncertainty about Taiwanese delicacies can be reduced through language translation and adjustments to message content and presentation. Therefore, message content and presentation (i.e., informational appeals), as a form of information dissemination, can be used as a tool for influencing consumers. Many studies have shown that message appeals can arouse consumers’ interest and persuade them to buy goods or services, which affects their attitudes and emotions and ultimately triggers specific purchase behaviours [14,15,16]. Therefore, it is necessary to understand how to use appropriate message appeals to influence foreigners’ perceptions of Taiwanese food tourism and their specific consumption behaviours.

In addition to the external environmental factors of informational appeals, their internal factors are worth investigating. Personality traits are unique to each individual and have a profound impact on the individual’s thoughts, feelings and behaviours. Personality traits are affected by important factors such as heritage and cultural background [15,17,18,19], which can influence the effect of the external environment [20,21]. Among the food-related personality traits, food neophobia and food neophilia are prominent. In addition to choosing appropriate message appeal models prior to marketing and promoting Taiwanese delicacies, it is necessary to attract potential customers by paying attention to tourists’ food neophobia and food neophilia traits.

Given that food neophobia and food neophilia are most relevant to food tourism, in this study, we analysed which of three message appeal schemes is the most suitable form of information to attract foreigners and serve as marketing and promotion for Taiwan’s catering and tourism industries. Previous studies on food neophobia/neophilia traits have mostly focused on Western and European foods and have rarely investigated the effect of message appeal on the consumption of traditional rice-based street food in Taiwan (e.g., PBC and meatballs). Therefore, this study used food neophobia/neophilia traits as moderator variables to explore their interaction with independent variables and their influence on foreigners’ willingness to eat Taiwanese street food.

## 2. Literature Review

### 2.1. Food Tourism

For tourists, the lure to experience a food culture vastly different from their own guides them to understand the local culture through food, food preparation and the dining environment and atmosphere. These activities provide them with a new and profound experience as well as lasting good memories of the tourism destination, which intensifies their experience of and feelings about the tourism destination [4,5,6,7]. Many studies of tourist destinations have shown that food tourism has various impacts on the local society, economy and environment [13]. For example, (1) it helps to promote the sustainability of the local economic environment and tourism industry, especially the promotion and revival of local foods, traditional foods and dietary traditions [22,23]; (2) it helps to preserve social and cultural heritage and memories and traditional lifestyles [7]; (3) it helps to develop regional identity and improve its visibility; and (4) it helps to strengthen the awareness and sustainable development of tourist destinations and the environment [24,25]. In the present study, food tourism refers to tourism that attracts foreigners to the region using the region’s characteristic cuisine and food culture as the main attraction [13]. In this study, Taiwanese delicacies were used as the focus of Taiwanese food tourism promotion.

### 2.2. Information Appeal

According to Albers-Miller and Stafford [26], rational appeal targets the information processing mode in the decision-making process to enable consumers to make logical and rational decisions. Message appeals attract consumers’ attention through attributes or characteristics unique to a product or service. Emotional appeals emphasize the creation of a situational experience at the emotional and experience levels of consumption; that is, emotional appeal stimulates consumers’ responses to a certain product or service and thus triggers their purchase intention. Therefore, for service or product suppliers, emotional appeal is a message transmission strategy that is designed to attract consumers’ attention [13]. Stienmetz et al. [27] noted that this strategy aims to create a positive link between a product or service and a situation to influence consumers’ perception of the product or service and thus their cognition. Based on the above studies, the present study defines rational appeal as an appeal made from the perspective of cognition and product characteristics that expresses information in terms of facts and data and emphasizes the function, quality and value of the product. In contrast, emotional appeal is an appeal to experience and emotion that expresses information in terms of senses, emotions and culture and emphasizes the creation of a beautiful perceptual atmosphere or emotion. According to de Souza et al. [28], when promoting a product or service with which consumers are not familiar, rational appeal should be adopted because consumers’ interest in the product or service can be cultivated through facts or data. Hwang and Lin [29] found that providing product information (e.g., nutrition information) increased the willingness of American consumers to consume Asian food because nutrition information can reduce consumers’ uncertainty about choosing unfamiliar foods. Rational appeal, which emphasizes the value of practicality, prompts and increases consumers’ willingness to eat food. In contrast, some scholars have argued that emotional appeal should be used as a communication strategy. Choi [30] found that the emotional appeal strategy should be adopted for all products and services because when consumers decide whether to purchase a product or service, they do not need a reasonable or logical reason and are often influenced by the content of the intended information, such as its convention, affiliation or particularity.

### 2.3. Food Neophilia and Neophobia Traits

Neophilia and neophobia are personality traits that indicate an individual’s attitude and behaviour towards new things. Although there are differences among individuals, neophilia refers to an individual’s tendency to approach or try new things out of interest or curiosity with the aim of seeking diversity, while neophobia is the opposite trait [31]. Food-related neophilia and neophobia traits are called food neophilia and food neophobia, respectively, and they play an extremely important role in food choices and preferences [5,32].

Food neophiles have a tendency to actively seek out and try unfamiliar foods and have a positive reaction to novel foods and exotic dishes due to their characteristics of curiosity and exploration [7,29,33]. Food neophobes not only do not seek out unfamiliar foods but also have a strong tendency to avoid, resist or even abhor unfamiliar foods; thus, they react negatively to novel foods and exotic dishes and regard them as dangerous and unsafe [5,17,29,33].

Food neophilia and food neophobia are food-related personality traits that refer to an individual’s thoughts, feelings, attitudes and behaviour patterns regarding food [34]. They affect individuals’ food-related attitudes and behaviours, and individuals may use them as selection and judgement criteria when faced with unfamiliar foods. For example, when faced with unfamiliar food, food neophiles choose to eat it out of interest or to satisfy their curiosity, while food neophobes strongly reject, resist and avoid it. Food neophilia and neophobia form and adjust individuals’ attitudes towards unfamiliar foods and their intention to eat them [35,36]. In the past, studies have mainly focused on the effect of internal psychological aspects on external behaviour and attitudes, such as individuals’ daily diet choices and preferences [16,37], and on predicting individuals’ intent to purchase unfamiliar foods [1,3,30,38]. In contrast, research has rarely addressed whether the interaction between the external environment-message appeal and food neophilia and neophobia traits affects or alters individuals’ willingness to eat unfamiliar foods.

Based on the above-described studies, we postulate that food neophiles have expectations regarding eating unfamiliar foods and are interested in foods that they have not tasted before, exotic dishes or ingredients, or cooking materials, methods or habits of food culture that are not generally accepted; therefore, they will be willing to try unfamiliar foods. In contrast, food neophobes are unwilling to eat unfamiliar foods; they do not accept or fear foods that they have not tasted, exotic dishes or ingredients, or cooking materials, methods or habits of food culture that are not generally accepted.

### 2.4. Eating Intention and Behaviour Intention

Lee et al. [39] demonstrated the profound effect of food neophilia/neophobia on predicting consumers’ willingness to try unfamiliar foods. Henriques et al. [40] also confirmed that food neophilia/neophobia affects consumers’ preference for novel foods and can be used to predict their acceptance of such foods. Both Pliner and Hobden [41] and Mascarello et al. [42] agreed that food neophobia is a manifestation of behaviour and personality and has been proven to effectively predict consumers’ willingness to eat unfamiliar foods. Mak et al. [43], Dimitrovski and Crespi-Vallbona [5] and Bondzi-Simpson and Ayeh [4] also showed that food-related personality traits are important physiological factors that affect travellers’ food consumption.

According to Casaló et al. [44], behavioural intention refers to the strength of an individual’s willingness to engage in a specific behaviour. It is highly positively correlated with the individual’s actual behaviour because it reflects the individual’s willingness to engage in or attitude towards engaging in a specific behaviour; this description is consistent with the views of Arvola et al. [45] and Bondzi-Simpson and Ayeh [4]. Ness et al. [46] stated that behavioural intention can be predicted through three items, i.e., the willingness to continue using a product, the willingness to recommend the product to relatives and friends and the willingness to use the product for special events and occasions. Therefore, in the present study, consumption willingness refers to the intensity of consumers’ willingness to eat specific foods and can be used to predict consumers’ willingness to consume a specific food or their attitude towards consuming it.

Based on the above literature review, we propose the following hypotheses:

**Hypothesis 1** **(H1).** *Message appeals affect foreigners’ willingness to eat Taiwanese street food.*

**Hypothesis 2** **(H2).** *Food neophilia/neophobia traits affect foreigners’ willingness to eat Taiwanese street food.*

**Hypothesis 3** **(H3).** *Food neophilia/neophobia traits have a moderating effect on the influence of message appeals on foreigners’ willingness to eat Taiwanese street food.*

## 3. Methods

### 3.1. Research Design

This study was designed to examine the influence of message appeals on foreigners’ willingness to eat Taiwanese street food and to determine the influence of message appeals on eating intention and whether message appeals have a moderating effect on the influence of food neophilia/neophobia on eating intention. Therefore, we adopted a quantitative experimental design and collected samples using snowball sampling [47]. Three groups, i.e., experimental group 1 (the rational appeal group), experimental group 2 (the emotional appeal group) and the control group (no appeal), were established.

Foreign tourists in Taiwan were recruited as the subjects and were randomly assigned to different experimental groups. Each group was administered a questionnaire survey through one of three online platforms, each of which presented a different situation. Links to the website containing the questionnaire survey were sent to each of the subjects through email (100 questionnaires), Facebook and Twitter (99 questionnaires), and each subject could choose the website link to which they responded. A total of 199 questionnaires were distributed, and 181 valid questionnaires were recovered, with a recovery rate of 91% [48]. The survey data were processed using the SPSS 22.0 and AMOS 6.0 statistical software packages.

### 3.2. Selection of Taiwanese Delicacies

The selection criteria for the two delicacies were as follows:

(A) Reports from both Taiwanese and international media.
(B) The delicacies’ appearance and ingredients. In terms of ingredients, both meatballs and PBC are rice-based, but they differ greatly in their production method, appearance, shape and taste and contrast one another.
(C) Pretests of the willingness-to-eat items involving five foreigners from the US, the UK, Italy, Turkey and Thailand who spoke English as their native language, an official language or a second/foreign language. The pretest aimed to ensure that foreigners’ willingness to eat the two Taiwanese street foods was consistent with expectations, i.e., that the meatballs were not controversial (i.e., participants were willing to eat them) while PBC was controversial (i.e., participants were unwilling to eat it).

### 3.3. Plot Design and Situational Manipulation Check

The message appeal was based on data collected from various websites, including the Tourism Bureau of the Ministry of Transportation of the Republic of China, Food Culture in Taiwan (taiwanfoodculture.net), international media reports (e.g., CNN, The New York Times, TIME and BBC), The Green Guide Taiwan, YouTube, newspapers and magazines. According to the operational definition applied for this study, the characteristics of Taiwanese street food were collated and ultimately classified into two categories, i.e., rational appeal and emotional appeal. Based on this information, a Chinese version of the collected data was generated. The Chinese version of the data was reviewed by three university professors of tourism and catering management who confirmed that it was in line with the operational definition used in this study; therefore, the Chinese text was used as the pilot in this study. After the pilot design was completed, the Chinese text was translated back into English and used to pretest five foreigners with English as their native language, official language, or foreign language. As a manipulation check of the tested experimental scenarios, the foreigners were asked to determine the type of appeal based on the content of the pilot.

### 3.4. Research Subject Selection

In this study, an experimental creation method was used to design the three scenarios. Because the research subjects were foreigners who met specific requirements, it was difficult to obtain samples; therefore, subjects who volunteered were selected using the snowball sampling method and were randomly assigned to different experimental groups. The questionnaire surveys were conducted through three online platforms, each of which had a dedicated scenario. Links to the websites hosting the questionnaire survey were sent to subjects through email, social network sites (Facebook, Twitter, blogs) and travel sites, among others, and the subjects could randomly choose from the three hosting webpages.

The subject requirements and selection criteria were as follows:

1. Participants from Europe or the United States. In other countries and regions in Asia (mainland China, Hong Kong, Japan and South Korea), the acceptance of food ingredients and food culture are similar to those in Taiwan, so subjects from these Asian countries were excluded.
2. Participants who spoke English as their native language or their first (or second) foreign language.
3. Foreigners who had never travelled to Taiwan for sightseeing, business, visiting relatives or friends, etc.
4. Foreigners who had visited Taiwan and stayed there for more than 24 h but not longer than 6 months for sightseeing, business, or visiting relatives or friends.
  (1) In addition to meeting requirements 1, 2 and 3, the subjects had to meet this requirement to avoid the acculturation phenomenon.
  (2) Acculturation refers to direct and continuous contact between groups of individuals from different cultural backgrounds that can cause a change in the original culture of one or both groups [49].

### 3.5. Questionnaire Design

The content of the questionnaire was based on Henriques et al. [40] and Ness et al. [46] and was reviewed by an expert group that included three university professors from the Department of Hospitality and Tourism, three government travel agency commissioners and three senior managers from travel agencies. It contained four parts in accordance with the research framework: (1) food neophilia/neophobia personality traits; (2) message appeal; (3) willingness to eat; and (4) basic information (e.g., gender, nationality, age, education, occupation, annual income, international travel frequency and sources of travel information). The items in Parts 1 and 3 were scored using a 5-level Likert scale (Strongly Disagree = 1; Disagree = 2; Neither Agree nor Disagree = 3; Agree = 4; Strongly Agree = 5). In Parts 2 and 3, each questionnaire showed pictures of the two street foods along with descriptions based on the designated type of message appeal. After reading the description and viewing the pictures, the respondents were asked to answer questions regarding their willingness to eat the delicacies.

## 4. Results and Discussion

### 4.1. Analysis of Basic Information and Tourist Information

Among the respondents, women were the majority, accounting for 53.6% of the total. In terms of age, respondents aged 21–25 years were the majority, accounting for 31.3%, followed by those aged 26–30 years, accounting for 22.1%. In terms of education level, those with a college or vocational college education were the majority, accounting for 60.8%, followed by those with a postgraduate education, accounting for 24.9%. In terms of occupation, students were the majority, accounting for 49.7%. In terms of region, respondents from North America were the majority, accounting for 39.8%, followed by those from Southeast Asia, accounting for 22.1%. In terms of the number of visited countries, most of the respondents had visited 1–5 countries, accounting for 45.9%, followed by those who had visited 6–10 countries, accounting for 29.8%. In terms of tourism information sources, most of the respondents (150 people) obtained tourism information from the Internet, followed by those who obtained information from relatives and friends (129 people) (Table 1).

### 4.2. Effect of Message Appeal on Willingness to Eat

Under different types of informational appeals, the foreign participants showed no difference in their willingness to eat meatballs (F = 0.25, *p* > 0.05). In other words, foreigners’ willingness to eat meatballs did not differ with different types of message appeal (Table 2).

However, foreigners’ willingness to eat PBC differed significantly according to the different types of message appeals (F = 8.67, *p* < 0.001) (Table 3).

### 4.3. Effect of the Food Neophilia/Neophobia Personality Trait on Willingness to Eat

Foreigners’ willingness to eat meatballs differed significantly between the two personality traits (food neophilia and food neophobia). Respondents with the food neophilia trait were more willing to eat meatballs than those with the food neophobia trait (Table 4).

Foreigners’ willingness to eat PBC also differed significantly between the two personality traits (food neophilia and food neophobia). Respondents with the food neophilia trait were more willing to eat PBC than those with the food neophobia trait (Table 4).

### 4.4. Hierarchical Regression Analysis

#### 4.4.1. Hierarchical Regression Analysis of the Effect of Food Neophilia/Neophobia Personality Traits, Message Appeal and Their Interaction on Foreigners’ Willingness to Eat Meatballs

To examine the moderating effect, the interaction variables (rational appeal-food neophilia/neophobia trait and emotional appeal*food neophilia/neophobia trait) were included in the regression equation. A moderating effect was found on foreigners’ food neophilia/neophobia trait and the willingness to eat meatballs, with a joint interpretation power of 21%. This finding validates the moderating effect and indicates that the interaction between food neophilia/neophobia traits and the message appeal type has a moderating effect on foreigners’ willingness to eat meatballs and that for foreigners with different food personality traits, different types of message appeals have different effects on their willingness to eat meatballs (Table 5). The street food selected in this study was pork products, and the respondents from West Asia were mostly Muslims. Because this study did not exclude the religious factors of Muslims, there was a significant effect of not eating pork products.

#### 4.4.2. Hierarchical Regression Analysis of the Effect of Food Neophilia/Neophobia Personality Traits, Type of Message Appeal and Their Interaction on Foreigners’ Willingness to Eat PBC

To examine the moderating effect, the interaction variables (rational appeal*food neophilia/neophobia trait and emotional appeal*food neophilia/neophobia trait) were included in the regression equation. The interaction variable had a significant predictive effect on foreigners’ willingness to eat PBC, with a joint interpretation power of 30%. This finding validates the moderating effect, indicating that the interaction between food neophilia/neophobia traits and message appeal has a moderating effect on foreigners’ willingness to eat PBC and that for foreigners with different food personality traits, different types of message appeals have different effects on their willingness to eat PBC (Table 6).

## 5. Conclusions and Discussion

### 5.1. Make Proper Use of Message Appeals to Influence Foreigners’ Acceptance of Taiwanese Street Food

The results of the test for the difference between means showed that the type of message appeal had a significant impact on foreigners’ willingness to eat PBC. Furthermore, the effect of the rational appeal (2.81) was significantly lower than that of the emotional appeal (3.22) or no appeal (3.59), indicating that with the rational appeal, foreigners were less willing to eat PBC and more inclined not to eat it. Based on this result, in terms of information appeal, displaying the product attributes and related facts and data about PBC (e.g., its English name (pig blood cake), ingredients (pig blood) and nutritional information) and thus directly conveying the true nature of PBC to foreigners is often inappropriate and inconsistent and tends to cause misunderstanding. Many dishes in different cuisines of the world use animal blood as an ingredient. The UK, France, Spain, Poland, Italy and other countries use pig blood, internal organs, rice, cereals and other ingredients to prepare distinctive dishes that have more implicit names, e.g., black pudding.

From the perspective of product sales, it is necessary to make product information public and transparent to consumers. However, from the perspective of food tourism, food culture is a tourist attraction that helps tourists understand the local culture through local foods and a unique dining environment and atmosphere. For tourists, the ultimate goal of touring a place is to feel content and thus to have positive behavioural and revisit intentions in the future and even to promote the destination to other people by word of mouth [4,5,39]. Therefore, when promoting Taiwanese street food to food tourists, it is recommended that the use of rational appeal be reduced or even avoided; instead, emotional methods that emphasize pictures, stories, emotions and cultural experiences should be adopted to help foreigners reduce their fear and uncertainty regarding certain Taiwanese delicacies.

### 5.2. Foreigners’ Food Neophilia/Neophobia Has a Significant Impact on Their Willingness to Eat Taiwanese Delicacies

The results of the test of differences between means showed that food neophilia/neophobia had a significant impact on foreigners’ willingness to eat Taiwanese street food. For both meatballs and PBC, food neophiles were more willing to try unfamiliar food than food neophobes. However, the effect was especially strong for the willingness to eat PBC; food neophiles were more inclined to eat it (3.62), while food neophobes were more inclined to not eat it (2.95). This effect can be examined from two aspects: the food neophilia/neophobia trait and PBC per se. The food neophilia/neophilia personality trait tends to stabilize with age. The results of this study are consistent with previous findings showing that the structure and formation of personality traits are affected by the external environment and cultural background in addition to intrinsic genetic factors, all of which prompt individuals to develop unique ideas, attitudes, emotional responses and behaviour patterns [42], i.e., the food neophilia/neophilia personality trait affects individuals’ food preferences and eating intentions [1,3,4,30,38]. In the food culture of most subjects in this study, dishes such as PBC that contain offal and blood ingredients are usually unacceptable. Therefore, when they were presented with strange foods that were unfamiliar to them or that deviated from their food cultures, the subjects were more profoundly affected by their food neophilia/neophilia: food neophiles approached the food with expectation, interest and curiosity and were more inclined to try it, while food neophobes avoided the food and were more inclined not to eat it.

### 5.3. Foreigners of Certain Nationalities Have a Tendency to Reject Certain Taiwanese Delicacies

Food initially existed for human beings only to meet the most basic physiological needs. Because it differed according to differences in climate, geography and human environment in various places, it developed cultural meanings that were exclusive to various places; as a result, food culture has representative and irreplaceable characteristics. People from different ethnic groups, nationalities, cultural backgrounds and social environments have developed unique ways of eating with different emphases. Harrington and Ottenbacher [50] and Mascarello et al. [42] revealed that although food tourism serves the same purpose across cultures, tourists from different countries and regions differ in the local foods they focus on at a given tourism destination. The main ingredients of meatballs and PBC, the two Taiwanese delicacies studied here, reflect Taiwan’s traditional customs and the early Taiwanese people’s thrifty lifestyle and hard life in the past. However, this cultural significance may not be meaningful to many foreigners, and cultural differences between visitors and locals can mean that Taiwanese street foods favoured by locals are not culturally accepted by some foreigners. The hierarchical regression analysis results indicated that certain nationalities had a negative predictive effect on the willingness to eat meatballs or PBC. Differences in the natural and humanistic environment have created various food cultures unique to each country or region. Therefore, when promoting food tourism or Taiwanese special dishes in the future, we should first fully understand the culture of the region (or country) to ensure that the promoted items conform to the national conditions and regional culture in a way that encourages effective promotion while avoiding the adverse effect of causing residents to reject the promoted items.

### 5.4. Food Neophilia/Neophobia Regulates the Effect of Message Appeals on Foreigners’ Willingness to Eat Taiwanese Delicacies

As an explicit personality trait, food neophilia/neophobia can be directly or indirectly detected by others. The hierarchical regression analysis results indicated that food neophilia/neophobia regulates the effect of message appeals on foreigners’ willingness to consume Taiwanese street food, which is consistent with previous studies’ findings that personality traits affect individuals’ acceptance of and response to message appeals [34,35]. We also found that for foreigners with different food neophilia/neophobia traits, message appeals had different effects on their willingness to eat Taiwanese delicacies. This is consistent with the finding of de Souza et al. [28] that personality traits affect individuals’ attitudes toward, perceptions of and behavioural response regarding the content of the information appeal; that is, even with the same or similar messages, different personality traits lead to different attitudes, perceptions and behaviours. Therefore, we postulate that compared with the external environmental impact of information appeal, the internalized food neophilia/neophobia trait has a greater influence on individuals’ behaviour patterns and intentions, which is in line with the conclusions of Nezlek and Forestell [37], Ji et al. [16] and Wolff and Larsen [7].

## 6. Contribution

Previous studies have investigated information appeal and food neophilia/neophobia traits. However, the influence of message appeals on the consumption of Taiwanese rice-based street foods and the moderating effect of the neophilia/neophobia trait on this influence have rarely been addressed. In this study, we demonstrated that message appeal types and food neophilia/neophobia traits have a significant impact on foreigners’ willingness to eat Taiwanese street food. This finding has implications for the Taiwanese catering and hospitality industries and government agencies when promoting Taiwanese food tourism and selecting marketing strategies and is the most significant contribution of this study.

## 7. Limitations

The subjects of this study were foreigners who met specific requirements. The sample selection required the use of snowball sampling and subsequent random assignment of the participants to experimental groups. The participants responded randomly to online questionnaires accessed through email, social networking sites, and travel sites. As a result of the characteristics of snowball sampling and the distribution of the online questionnaires through social media websites, the occupations and nationalities of the respondents were unevenly represented. This uneven distribution of participant characteristics made it impossible to conduct a more in-depth study of people from certain countries or regions. To avoid content bias and excessive questionnaire length, only two common Taiwanese street foods, meatballs and PBC, were chosen for consideration in this study. Both delicacies are rice-based and were considered in a pretest; however, they may still cause aversions among certain ethnic groups, which may have biased our conclusions. In the future, other Taiwanese street foods with different ingredients will be used to avoid such bias.

## Figures and Tables

**Table 1:** Demographic characteristics (*n* = 181).

Characteristics	N	Percentage	Characteristics	N	Percentage
**Gender**			**Education**		
Male	84	46.4	High school, vocational school	26	14.4
Female	97	53.6	College	110	60.8
**Age**			Graduate school	45	24.9
15–20	37	20.4			
21–25	57	31.6	**Occupation**		
26–30	40	22.1	Agriculture, fisheries and manufacturing	7	3.9
31–35	19	10.5	Service industry	11	6.1
36–40	28	15.4	Other	73	40.3
**Nationality**			**Number of countries visited**		
Southeast Asian	40	22.1	Have not been to other countries	15	8.3
New Zealander or Australian	10	5.5	1–5 countries	83	45.9
European	7	3.9	6–10 countries	54	29.8
West Asian	35	19.3	11–15 countries	10	5.5
North American	72	39.8	16–20 countries	10	5.5
Central or South American	11	6.1	More than 21 countries	9	5.0
Other	6	3.3			

Note: West Asia includes Turkey, Cyprus, Syria, Lebanon, Iraq, Iran, Israel, Jordan, Egypt, Sudan, Libya and the Arabian Peninsula countries.

**Table 2:** Effect of message appeal type on foreigners’ willingness to eat meatballs (*n* = 181).

Variable	Message Appeal	Number of People	Mean	Standard Deviation	Source of Variation	df	MS	F	Sig.
Willingness to eat meatballs	No appeal	50	3.67	0.59	Inter-group	2.00	0.11	0.25	0.78
	Rational appeal	61	3.58	0.74	Intra-group	178.00	0.46		
	Emotional appeal	70	3.61	0.67	Total	180.00			

**Table 3:** Effect of message appeal on foreigners’ willingness to eat PBC (*n* = 181).

Variable	Message Appeal	Number of People	Mean	Standard Deviation	Source of Variation	df	MS	F	Sig.	Scheffe
Willingness to eat PBC	No appeal	50	3.59	0.82	Inter-group	2.00	8.40	8.67	0.00 ***	1 > 3 > 2
	Rational appeal	61	2.81	1.12	Intra-group	178.00	0.97			
	Emotional appeal	70	3.22	0.97	Total	180.00				

Note: *** *p* < 0.001.

**Table 4:** Effect of food-related personality traits on foreigners’ willingness to eat meatballs (*n* = 181).

Variable	Food-Related Personality Trait	Number of People	Mean	Standard Deviation	t	df	Sig. (2-Tailed)
Willingness to eat meatballs	Neophilia	64	3.80	0.48	3.13	174	0.00 **
	Neophobia	117	3.52	0.74			
Willingness to eat PBC	Neophilia	64	3.62	1.04	4.43	179	0.00 ***
	Neophobia	117	2.95	0.94			

Note: ** *p* < 0.01; *** *p* < 0.001.

**Table 5:** Hierarchical regression analysis of the effect of food neophilia/neophobia personality traits, message appeal type and their interaction on foreigners’ willingness to eat meatballs.

		Block 1	Block 2	Block 3	Block 4	Block 5	Block 6
Variables in the Model		Beta	Beta	Beta	Beta	Beta	Beta
*Demographic variables*	Gender	0.15	0.05	0.06	0.08	0.05	0.03
	Age	−0.06	−0.07	−0.07	−0.08	−0.08	−0.08
	Educational level (college as benchmark)						
	High school, vocational school, or below	−0.23	−0.28	−0.27	−0.23	−0.24	−0.21
	Graduate school	−0.24	−0.24 *	−0.24 *	−0.28 *	−0.27 *	−0.26 *
	Occupation (student as benchmark)						
	Agriculture, fisheries and manufacturing	0.39	0.34	0.35	0.39	0.38	0.34
	Service industry	−0.15	−0.21	−0.21	−0.20	−0.18	−0.18
	Other	0.00	0.03	0.02	0.03	0.00	−0.01
	Nationality (North American as benchmark)						
	Southeast Asian		−0.14	−0.18	−0.16	−0.08	−0.05
	New Zealander or Australian		−0.04	−0.04	0.01	−0.01	−0.02
	European		−0.05	−0.04	−0.09	−0.10	−0.11
	West Asian		−0.81 ***	−0.80 *	−0.85 *	−0.80 ***	−0.81 ***
	Central or South American		−0.13	−0.14	−0.14	−0.07	−0.08
	Other		−0.47	−0.49	−0.47	−0.34	−0.30
	Number of countries visited			−0.03	−0.02	0.00	−0.01
	Number of Asian countries visited			0.02	0.02	0.01	0.01
	Message appeal type (no appeal as benchmark)						
	Rational appeal				−0.24	−0.21	0.02
	Emotional appeal				−0.13	−0.08	−0.03
	Food neophilia/neophobia					−0.25 *	−0.09
	Rational appeal*Food neophilia/neophobia trait						−0.39
	Emotional appeal*Food neophilia/neophobia trait						−0.10
*Model summary*	*R* ^2^	0.08	0.16	0.16	0.17	0.20	0.21
	*F*	2.13 *	2.37 **	2.07 *	2.03 *	2.24 **	2.15 **
	∆*R*^2^	0.08	0.08	0.00	0.02	0.02	0.01
	*F* change	2.13 *	2.52 *	0.26	1.57	4.97 *	1.27

Dependent variable: willingness to eat meatballs. Note: * *p* < 0.05; ** *p* < 0.01; *** *p* < 0.001.

**Table 6:** Hierarchical regression analysis of the effect of food neophilia/neophobia personality traits, types of message appeals and their interaction on foreigners’ willingness to eat PBC.

		Block 1	Block 2	Block 3	Block 4	Block 5	Block 6
Variables in the Model		Beta	Beta	Beta	Beta	Beta	Beta
*Demographic variables*	Gender	−0.11	−0.11	−0.10	−0.06	−0.09	−0.09
	Age	0.07	0.06	0.07	0.05	0.04	0.03
	Education level (college as benchmark)						
	High school, vocational school, or below	−0.11	−0.14	−0.13	−0.10	−0.11	−0.13
	Graduate school	−0.13	−0.14	−0.13	−0.19 *	−0.18 *	−0.16 *
	Occupation (student as benchmark)						
	Agriculture, fisheries and manufacturing	−0.02	−0.05	−0.05	−0.03	−0.04	−0.04
	Service industry	−0.10	−0.15	−0.15	−0.15	−0.13	−0.14
	Other	−0.10	−0.15	−0.16	−0.15	−0.19	−0.19 *
	Nationality (North American as benchmark)						
	Southeast Asian		−0.26 *	−0.29 *	−0.28 *	−0.20 *	−0.21 *
	New Zealander or Australian		−0.08	−0.08	−0.05	−0.06	−0.05
	European		0.06	0.07	0.00	−0.01	0.00
	West Asian		−0.07	−0.07	−0.10	−0.08	−0.06
	Central or South American		−0.05	−0.06	−0.05	−0.02	−0.01
	Other		−0.14	−0.14	−0.14	−0.08	−0.10
	Number of countries visited			−0.07	−0.03	0.01	0.00
	Number of Asian countries visited			0.07	0.10	0.01	0.02
	Message appeal (no appeal as benchmark)						
	Rational appeal				−0.35 ***	−0.32 ***	−0.30 *
	Emotional appeal				−0.24 *	−0.18	0.07
	Food neophilia/neophobia trait					−0.28 ***	−0.13
	Rational appeal*Food neophilia/neophobia trait						−0.03
	Emotional appeal*Food neophilia/neophobia trait						−0.35 *
*Model summary*	*R* ^2^	0.04	0.13	0.14	0.21	0.27	0.30
	*F*	1.13	1.97 *	1.74 *	2.50 *	3.32 ***	3.38 ***
	∆*R*^2^	0.04	0.09	0.00	0.07	0.06	0.03
	*F* change	1.13	2.88	0.30	7.27 ***	13.89 ***	3.11 *

Dependent variable: willingness to eat PBC. Note: * *p* < 0.05; *** *p* < 0.001.

## Data Availability

Not applicable.
